# Investigation of Perovskite Oxide SrCo_0.8_Cu_0.1_Nb_0.1_O_3–*δ*_ as a Cathode Material for Room Temperature Direct Ammonia Fuel Cells

**DOI:** 10.1002/cssc.201900451

**Published:** 2019-05-22

**Authors:** Peimiao Zou, Shigang Chen, Rong Lan, Shanwen Tao

**Affiliations:** ^1^ School of Engineering University of Warwick Coventry CV4 7AL UK; ^2^ Department of Chemical Engineering Monash University Clayton Victoria 3800 Australia; ^3^ Faculty of Engineering, Environment & Computing Coventry University Coventry CV1 5FB UK

**Keywords:** alkaline, cathode, direct ammonia fuel cell, non-precious metal catalyst, perovskite oxide

## Abstract

Single‐phase perovskite oxide SrCo_0.8_Cu_0.1_Nb_0.1_O_3–*δ*_ was synthesized using a Pechini method. X‐ray diffraction (XRD) analysis indicated a cubic structure with *a*=3.8806(7) Å. The oxide material was combined with active carbon, forming a composite electrode to be used as the cathode in a room temperature ammonia fuel cell based on an anion membrane electrolyte and NiCu/C anode. An open circuit voltage (OCV) of 0.19 V was observed with dilute 0.02 m (340 ppm) ammonia solution as the fuel. The power density and OCV were improved upon the addition of 1 m NaOH to the fuel, suggesting that the addition of NaOH, which could be achieved through the introduction of alkaline waste to the fuel stream, could improve performance when wastewater is used as the fuel. It was found that the SrCo_0.8_Cu_0.1_Nb_0.1_O_3−*δ*_ cathode was converted from irregular shape into shuttle‐shape during the fuel cell measurements. As the key catalysts for electrode materials for this fuel cell are all inexpensive, after further development, this could be a promising technology for removal of ammonia from wastewater.

## Introduction

Fuel cells are a promising technology for converting the chemical energy in fuels directly into electrical energy. Ammonia is an important energy conversion and hydrogen storage material with a hydrogen weight percent of 17.8 wt %.^1^, ^2^ Compared with other fuels, such as hydrogen, methanol, and ethanol, ammonia has been regarded as a promising potential fuel because it is carbon‐free with high volumetric and gravimetric energy densities.^2^, ^3^ In addition, there is a high concentration of ammonia in wastewater, such as sewage, landfill leachate, and spent lee from distilleries. Sewage wastewater may also contain urea; however, urea naturally hydrolyses into ammonia,^4^ and there are free ammonia or ammonium ions existing in wastewater.^5^ Conventionally, ammonia removal is done using a combined aerobic and anaerobic treatment process in wastewater treatment plants (WWTPs). This process requires energy for aeration, which can constitute up to 60 % of the total energy consumption of a WWTP.^6^ On the other hand, ammonia is a typical fuel that contains a large quantity of chemical energy. Instead of consuming energy, useful electricity can be generated from ammonia in wastewater if a low‐cost ammonia fuel cell is developed and applied; ammonia fuel cells are promising technologies to remove ammonia in wastewater at low or negative energy consumption.

Recently, ammonia fuel cells have attracted researchers’ interests.^7^, ^8^, ^9^ The main reports on ammonia fuel cells are based on solid oxide electrolytes. The high operating temperature of ammonia solid oxide fuel cells (SOFCs) or ammonia alkaline fuel cells (AFCs) makes them very difficult to be directly used for ammonia‐containing wastewater treatment owing to the high thermal capacity of water.^7^, ^10^, ^11^, ^12^ There are also reports on low‐temperature ammonia fuel cells based on alkaline membrane or acidic Nafion membrane electrolytes.^5^, ^13^, ^14^, ^15^ In reported room temperature ammonia fuel cells, precious metals, such as Pt, were regarded as one of the most active electrocatalysts for the ammonia oxidation reaction (AOR) and oxygen reduction reaction (ORR) owing to their high activity and low overpotential.^7^, ^13^, ^14^, ^16^, ^17^, ^18^, ^19^, ^20^ A direct ammonia microfluidic fuel cell was also reported using KOH solution as the electrolyte.^21^

The key to improving the working efficiency of ammonia fuel cells is to improve the performance of the catalysts in the anode, cathode, and electrolyte, and to reduce the overall cost. One of the key challenges towards this goal is to develop low‐cost electro‐catalysts. In our research, we focused on developing a new low‐cost electrocatalyst to improve performance of room temperature ammonia fuel cells under alkaline conditions.

In an ammonia fuel cell, AOR occurs at the anode:^5^

(1) $$2\;\mathrm{NH}{}_{3}+6\;\mathrm{OH}{}^{-}\to N{}_{2}+6\;H{}_{2}O+6\;e{}^{-}\;\;\;E{}^{0}=-0.77\;V,$$

and ORR occurs at the cathode:^5^, ^22^

(2) $$O{}_{2}+2\;H{}_{2}O+4\;e{}^{-}\to 4\;\mathrm{OH}{}^{-}\;\;\;\;E{}^{0}=+0.40\;V.$$

At present, many non‐precious metal catalysts with higher activity for ORR have been discovered to replace Pt group metals. For example, metal/nitrogen/carbon composites prepared by the pyrolysis of inexpensive materials can effectively control the porosity and final structure of the catalysts. In addition, ORR is very sensitive to surface electronic properties and coordination of electron surface atoms or catalysts. Therefore, changing the metal organic framework and optimizing the catalyst atomic structure^23^ has also become a popular research area. For instance, heterogeneous atom‐doped carbon materials have been widely studied because of their low‐cost and abundant raw materials, high catalytic activity, high chemical stability, and environmental friendliness.^24^ Moreover, porous morphology and larger electrochemical surface area also improve catalytic activity.^25^ The best combination of oxide and nanocarbon can achieve excellent stability. In recent years, perovskite oxides have become a highly efficient ORR catalyst to replace precious metal catalysts because of their high catalytic activity, variety, and low cost.^26^

Perovskite oxides have been widely used as both cathode and anode in SOFCs. It is reported that mixed ionic and electronic conducting La_*x*_Sr_1−*x*_Co_1−*y*_Fe_*y*_O_3−*δ*_ (LSCF) perovskites are currently the adopted ORR cathode in industry.^27^ Other mixed conducting perovskite or perovskite‐related oxides, such as La_*x*_Sr_1−*x*_CoO_3−*δ*_ 28 and SrCo_1−*x*_Nb_*x*_O_3−*δ*_ 29, have better ORR activity than LSCF but have higher thermal expansion coefficients.^27^ From our previous work, we investigated Cu‐doped SrFe_0.9_Nb_0.1_O_3−*δ*_ and found that SrFe_0.8_Cu_0.1_Nb_0.1_O_3−*δ*_ exhibits the highest conductivity in air when applied in SOFCs.^30^ Therefore, the Cu‐doped SrCo_1−*x*_Nb_*x*_O_3−*δ*_, which was not reported before, is envisaged to also exhibit good ORR activity in fuel cells and could be a potential cathode material for room temperature fuel cells.

To the best of our knowledge, reports on using perovskite oxide as the cathode material in a two‐electrode fuel cell is scarce. In this study, we report the performance of a SrCo_0.8_Cu_0.1_Nb_0.1_O_3−*δ*_ cathode in a two‐electrode room temperature direct ammonia fuel cell.

A good AOR anode is also desired to rigorously investigate with the developed cathode material. There is a significant amount of research on the hydrogen oxidation reaction (HOR), which has similar reactions to AOR in alkaline medium.^31^ Identifying non‐Pt electrocatalysts for HOR and AOR is a challenge.^31^, ^32^ In our previous studies, we identified that NiCu bimetal and hierarchical nickel–copper hydroxide nanowires are excellent catalysts for electrochemical oxidation of ammonia.^33^, ^34^ These materials are expected to be good anode materials for ammonia fuel cells. Indeed, it was reported that NiCu nanoparticles supported on carbon is a good anode for a direct ammonia microfluidic fuel cell.^21^ Therefore, in this work, the NiCu/C catalyst is also used as an anode for a conventional ammonia fuel cell. Additionally, it was found that Ni‐based electrocatalysts exhibit high activity for hydrogen oxidation reaction (HOR);^35^ specifically, Ni_95_Cu_5_‐alloy nanoparticles supported in carbon blacks were successfully applied in an anion exchange membrane fuel cell.^36^ The key task of this work is to identify low‐cost oxide cathode materials for room temperature direct ammonia fuel cells. The fuel cell performance of a room temperature ammonia fuel cell containing a SrCo_0.8_Cu_0.1_Nb_0.1_O_3−*δ*_/C cathode and NiCu/C anode is presented.

## Experimental Section

### Synthesis of SrCo_0.8_Cu_0.1_Nb_0.1_O_3−*δ*_ and NiCu

The perovskite oxide SrCo_0.8_Cu_0.1_Nb_0.1_O_3−*δ*_ was synthesized through a Pechini method,^37^ similar to the synthesis of SrFe_0.8_Cu_0.1_Nb_0.1_O_3−*δ*_.^30^ All the chemical precursors, ammonium niobate(V) oxalate hydrate, C_4_H_4_NNbO_9_⋅*x* H_2_O (99.99 %, Sigma Aldrich, *x*=6.7), Sr(NO_3_)_2_ (98 %, Alfa Aesar), Co(NO_3_)_2_⋅6 H_2_O (97.7 % min, Alfa Aesar), and Cu(NO_3_)_2_⋅2.5 H_2_O (98 %, Alfa Aesar), were directly dissolved in deionized water to prepare a mixed solution. The stoichiometric molar ratio of C_4_H_4_NNbO_9_⋅*x* H_2_O, Sr(NO_3_)_2_, Co(NO_3_)_2_⋅6 H_2_O, and Cu(NO_3_)_2_⋅2.5 H_2_O with molar ratio of 0.1:1.0:0.8:0.1 was used. Then, citric acid (99+%, Alfa Aesar), with a molar ratio of 1.2:1 of citric acid to the total molar of metal ions, was added into the mixed solution. Ethylene glycol (Fisher Scientific), with a molar ratio of 1:1 to the citric acid, was added into the mixed solution and magnetically stirred at 120 °C for over 10 h on a hot plate. While stirring, a gel formed. Then the formed gel was dried at a constant temperature of 410 °C to be ignited for combustion. After the organic components in the mixture burned off during the drying process, the powder was ground and calcined in a muffle furnace at 500 °C for 2 h with the heating/cooling rate of 5 °C min^−1^. After regrinding, the powder was then fired at 1000 °C for 4 h with a heating/cooling rate of 3 °C min^−1^. The as‐prepared SrCo_0.8_Cu_0.1_Nb_0.1_O_3−*δ*_ was used for materials characterization and fuel cell measurements.

The NiCu nanoparticles supported on carbon black were synthesized by a hydrothermal method,^38^ similar to the method for preparation of Ni_50_Cu_50_/C catalyst reported in a previous paper.^21^ Firstly, carbon black (50 mg, Cabot Vulcan XC‐72R) was ultrasonicated in 5 mL deionized water for 30 min to form an ink. Then, NiSO_4_⋅6 H_2_O (0.114 g) and CuSO_4_⋅5 H_2_O (0.099 g) were added to the prepared ink and ultrasonicated for another 20 min. The mixture was placed in an ice‐water container. Fresh NaBH_4_ (0.1 g) solution, dissolved in 5 mL deionized water, was added dropwise into the container under stirring for 2 h. After reaction, the mixture was transferred to a Teflon‐lined stainless autoclave that was then sealed and put in an oven at 150 °C for 4 h. After cooling to room temperature, the powder was centrifuged at 10 000 rpm. for 10 min and washed several times with deionized water. The final NiCu/C catalysts were collected after drying at 70 °C for 12 h.

### Materials characterization

X‐ray diffraction (XRD) was carried out to identify the crystal structures on a Panalytical X′Pert Pro Multi‐Purpose Diffractometer (MPD) with CuKα1 radiation working at 45 kV and 40 mA. Absolute scans in the 2 *θ* range of 10–90° with step sizes of 0.0167° were used during data collection. Scanning electron microscopy (SEM) observation of the microstructure was carried out on a Zeiss SUPRA 55‐VP scanning microscope. Energy dispersive X‐ray spectroscopy (EDS) was used to analyze the electrode and determine the element composition of the samples, including both point and area analyses.^39^

### Fuel cell fabrication

Plain carbon fiber cloth (0.35 mm thickness, E‐TEK) was used as the substrate for the catalysts. The carbon cloth electrode (1×2 cm^2^) was sonicated in dilute hydrochloric acid, water, and isopropanol, respectively, as pretreatments. 1 g prepared SrCo_0.8_Cu_0.1_Nb_0.1_O_3–*δ*_ powder was mixed with 0.2 g carbon black (Cabot Vulcan XC‐72) and 0.2 g Amberlite IRA‐402(OH) resin (Alfa Aesar) through ball‐mill machine (Ortoalresa OABM 255) at 200 rpm for 24 h. Then polytetrafluoroethylene (PTFE) suspension with 0.2 g PTFE was added into the milled mixture using 5 mL water and 5 mL isopropanol as the solvent.^40^, ^41^ The mixture was stirred at room temperature for 48 h to prepare the ink. Afterwards, the as‐prepared ink was brushed onto the pre‐treated carbon cloth and the electrode was put in a fume cupboard to naturally dry overnight. The loading of catalyst SrCo_0.8_Cu_0.1_Nb_0.1_O_3–*δ*_ was approximately 18.67 mg cm^−2^. In fuel cell tests, a 1×1 cm^2^ cathode was used. The thickness of the SrCo_0.8_Cu_0.1_Nb_0.1_O_3–*δ*_/C catalyst layer was approximately 0.2–0.3 mm.

The fumapem FAA anion exchange membrane (Fumatech, OH^‐^form) was used as the electrolyte. The anode material was NiCu nanoparticles supported on carbon black, which was synthesized by hydrothermal method, as described above. Plain carbon cloth was also used as the anode substrate.^42^ The loading of NiCu/C catalyst was 2.5 mg cm^−2^ with the NiCu bimetal loading of 1.2 mg cm^−2^. The effective area of cell was 1×1 cm^2^.

### Electrochemical measurements

After all the components of fuel cell, including anode, cathode, and electrolyte membrane, were prepared, the fuel cell was assembled. Ammonia solution was slowly pumped into the anode chamber as the fuel, and compressed air was introduced into the cathode chamber from the opposite direction. The flow rate of ammonia solution was controlled by a small pump that rotated at 20 rpm with a flow rate of the ammonia solution of approximately 1 mL min^−1^. The flow rate of air was controlled at 10 mL min^−1^. The performance of the fuel cell was tested by a Solartron 1287A electrochemical interface controlled by electrochemical software CorrWare/CorrView.

The fuel was prepared using concentrated ammonia solution (35 %, 0.88 g mL^−1^, Fisher Chemical) and deionized water. NaOH (98 %, Alfa Aesar) was added to research the performance of fuel cell under alkaline conditions. At the beginning, the performance of the fuel cell was tested at different concentrations of ammonia solution at room temperature without the addition of NaOH. Then 1 m NaOH was added to the ammonia solution to adjust the pH value of the fuel. As the ammonia content in the wastewater is usually between 200 ppm (≈0.01 m) and 2000 ppm (≈0.1 m), it is important to test the performance of the cell when low concentration of ammonia solution is used as the fuel.

## Results and Discussion

### Characterization of the catalyst

**Figure 1:**
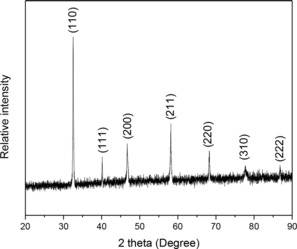
XRD pattern of the prepared SrCo_0.8_Cu_0.1_Nb_0.1_O_3−*δ*_ collected at room temperature.

XRD was used to determine the phase of the synthesized SrCo_0.8_Cu_0.1_Nb_0.1_O_3−*δ*_. The room temperature XRD pattern of the as‐prepared SrCo_0.8_Cu_0.1_Nb_0.1_O_3−*δ*_ powder is shown in Figure 1, confirming that a single‐phase perovskite oxide SrCo_0.8_Cu_0.1_Nb_0.1_O_3−*δ*_ was obtained. It can be indexed as a cubic structure with *a*=3.8806(7) Å. Therefore, a new perovskite oxide SrCo_0.8_Cu_0.1_Nb_0.1_O_3−*δ*_ was successfully synthesized.

### Ammonia fuel cell performance

#### Ammonia fuel cell performance without the addition of NaOH

In reported work on room temperature ammonia fuel cells, the addition of alkaline solutions, such as aqueous NaOH and KOH, increases the pH value, facilitating the oxidation of ammonia because of the increase in the OH^−^ ion concentration.^13^

The addition of NaOH/KOH will help the removal of ammonia, but it is desired to neutralize the alkaline wastewater in the following step before disposing to drainage, which means additional cost. Therefore, it would be ideal for the fuel cell to remove ammonia from wastewater without requiring alkaline additives. In this study, we initially measure the ammonia fuel cell performance using ammonia solution without addition of alkaline.

**Figure 2:**
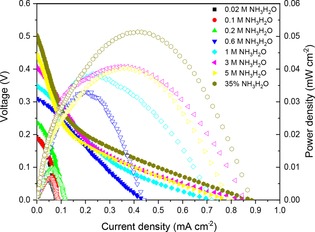
The room temperature fuel cell performance for an ammonia fuel cell at different ammonia concentrations using the SrCo_0.8_Cu_0.1_Nb_0.1_O_3−*δ*_ electrode as the cathode.

Figure 2 shows the current–voltage (*I*–*V*) curves of the fuel cell at room temperature with different concentrations of ammonia aqueous solution. It was found that the open circuit voltage (OCV) increased when the ammonia concentration increased. A maximum current and power density of 0.9 mA cm^−2^ and 0.053 mW cm^−2^ were achieved when 35 wt % concentrated ammonia was used as the fuel.

It should be noted that, when researchers investigate the activities of perovskite oxides on ORR in alkaline conditions, addition of a strong base, such as KOH or NaOH is very common to create an alkaline environment.^26^ However, from Figure 2, reasonable performance is achieved despite the absence of NaOH, especially at the lowest ammonia concentration of 0.02 m where an open circuit voltage (OCV) of 0.19 V is achieved. These results suggest that, for normal wastewater containing 1000 ppm (i.e., 0.06 m) ammonia, this fuel cell can generate almost 0.19 V OCV and 0.08 mA cm^−2^ current density without requiring the extra cost for increasing the pH value of the fuel, which is very promising for real applications.

#### Ammonia fuel cell performance with the addition of 1 m NaOH

In our previous studies, it was found that the NiCu bimetal and nickel copper hydroxide catalysts work better for AOR under alkaline conditions.^33^, ^34^ On the other hand, most perovskite oxides work better under alkaline conditions as ORR catalysts when measured by three electrode methods.^26^ Therefore, the addition of alkaline additives, such as NaOH, will increase the pH value and facilitate both the anode and cathode reactions, thus the ammonia fuel cell is expected to perform better.

**Figure 3:**
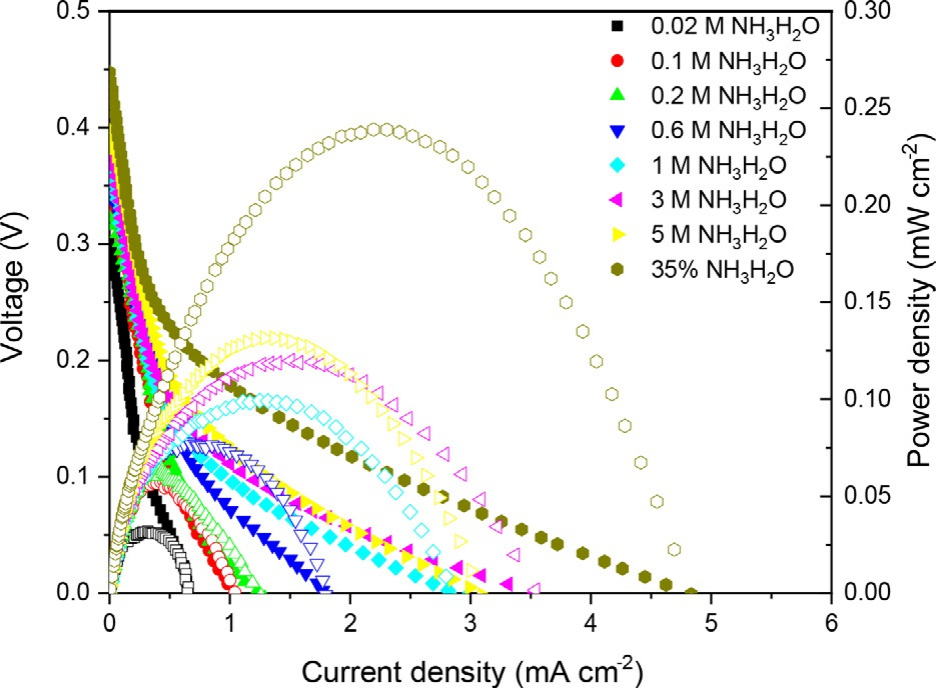
The room temperature fuel cell performance for an ammonia fuel cell at different ammonia concentrations with the addition of 1 m NaOH using the SrCo_0.8_Cu_0.1_Nb_0.1_O_3−*δ*_ electrode as the cathode.

Figure 3 shows the fuel cell performance when 1 m NaOH was added to the ammonia fuel. Compared with Figure 2, it is obvious that, when the ammonia concentration is lower than 1 m, adding NaOH to increase the pH of fuel can improve the OCV to 0.35 V and can increase the current density over 5 times. For high concentration ammonia, the increase of pH does not contribute the OCV, but can also improve current density and power density almost 7 times. A maximum current and power density of 5 mA cm^−2^ and 0.25 mW cm^−2^ was achieved when 35 wt % concentrated ammonia with 1 m NaOH was used as the fuel. This is because the concentration of OH^−^ ion is high when the ammonia concentration is high, resulting in a very high pH value of the fuel, which can increase the reaction rates of both AOR and ORR.

To quickly remove ammonia from wastewater, increasing the pH value of the fuel is a good choice. This can be achieved by adding alkaline waste, such as coal fly ash. The power density of the room temperature fuel cell is lower than those reported for hydrogen and hydrazine when Pt‐ or Pd‐based catalysts were used in the electrodes,^43^ but it is more suitable for large scale application because of the low‐cost catalysts used in this study.

### XRD and SEM/EDS analysis after ammonia fuel cell measurements

To study the stability of the cathode material under the fuel cell condition, XRD analyses were carried out on the cathode before after the fuel cell measurements.

**Figure 4:**
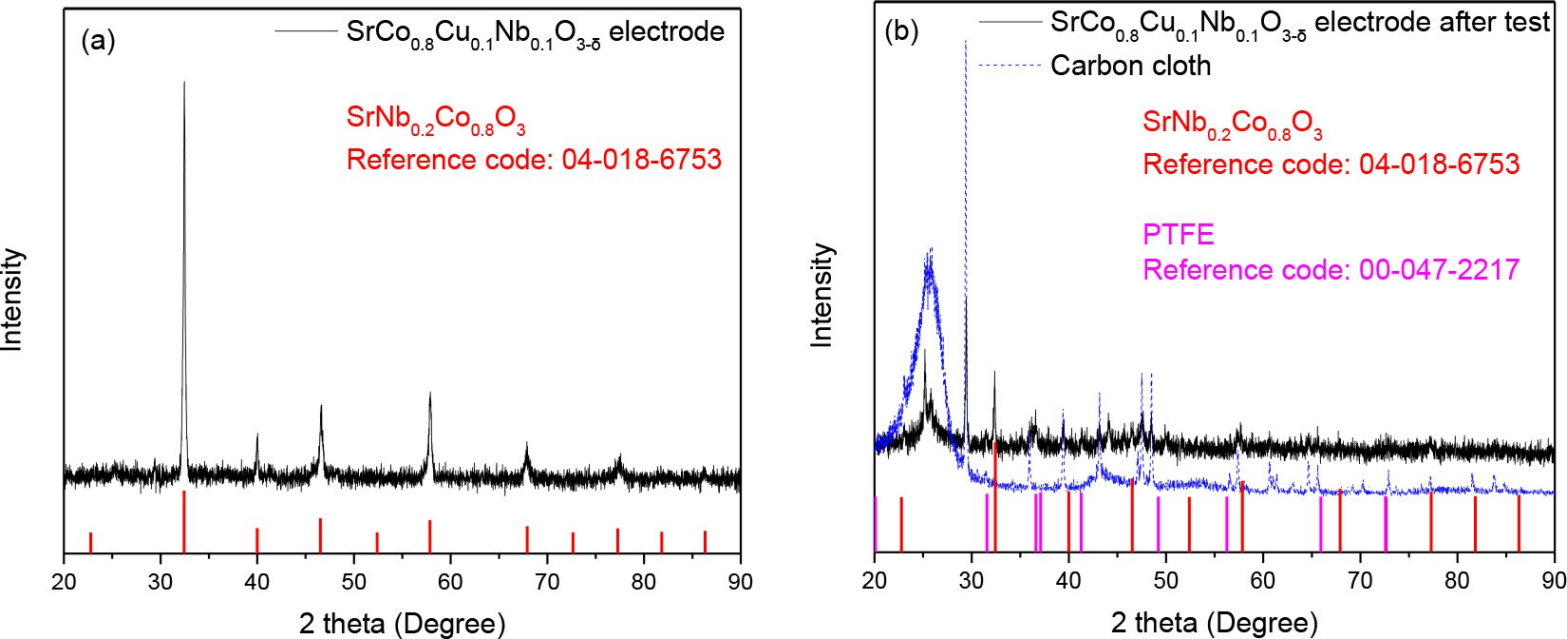
XRD data of the SrCo_0.8_Cu_0.1_Nb_0.1_O_3−*δ*_ cathode a) before and b) after the fuel cell measurements.

Figure 4 shows the XRD results of SrCo_0.8_Cu_0.1_Nb_0.1_O_3−*δ*_ electrode before (Figure 4 a) and after a long‐time test (Figure 4 b) at room temperature. The peaks of perovskite oxides can be observed easily in these two plots. Some obvious peaks, for example at 2 *θ* of ≈26° and 29°, of the electrode after the test are affected by the carbon cloth as shown in Figure 4 b. After the long‐time test, the majority of SrCo_0.8_Cu_0.1_Nb_0.1_O_3−*δ*_ on the carbon cloth electrode was stuck to the membrane, which affected the observation and research on the electrode. The small peak at 2 *θ* of ≈37° belongs to the PTFE binder.^44^ It was found that the intensity of the perovskite oxide SrCo_0.8_Cu_0.1_Nb_0.1_O_3−*δ*_ decreased after the fuel cell measurements. This could be related to the quantity of the powder because most catalyst was stuck on the alkaline when separating the membrane electrode assembly (MEA). Another possibility is that, the SrCo_0.8_Cu_0.1_Nb_0.1_O_3−*δ*_ became less crystallized. There could be some interaction between ammonia and/or NaOH with the SrCo_0.8_Cu_0.1_Nb_0.1_O_3−*δ*_ crystals, weakening the bonds in SrCo_0.8_Cu_0.1_Nb_0.1_O_3−*δ*_, resulting in reduced crystallinity. This is also confirmed by the SEM observation of the cathode, as discussed below.

**Figure 5:**
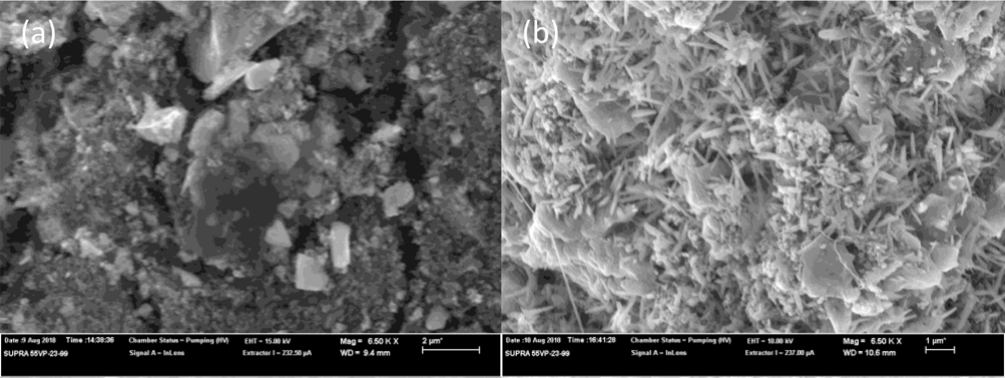
SEM results of the SrCo_0.8_Cu_0.1_Nb_0.1_O_3−*δ*_ electrode at 6500X a) before and b) after the fuel cell measurements. Scale bars correspond to 2 μm (a) and 1 μm (b).

**Figure 6:**
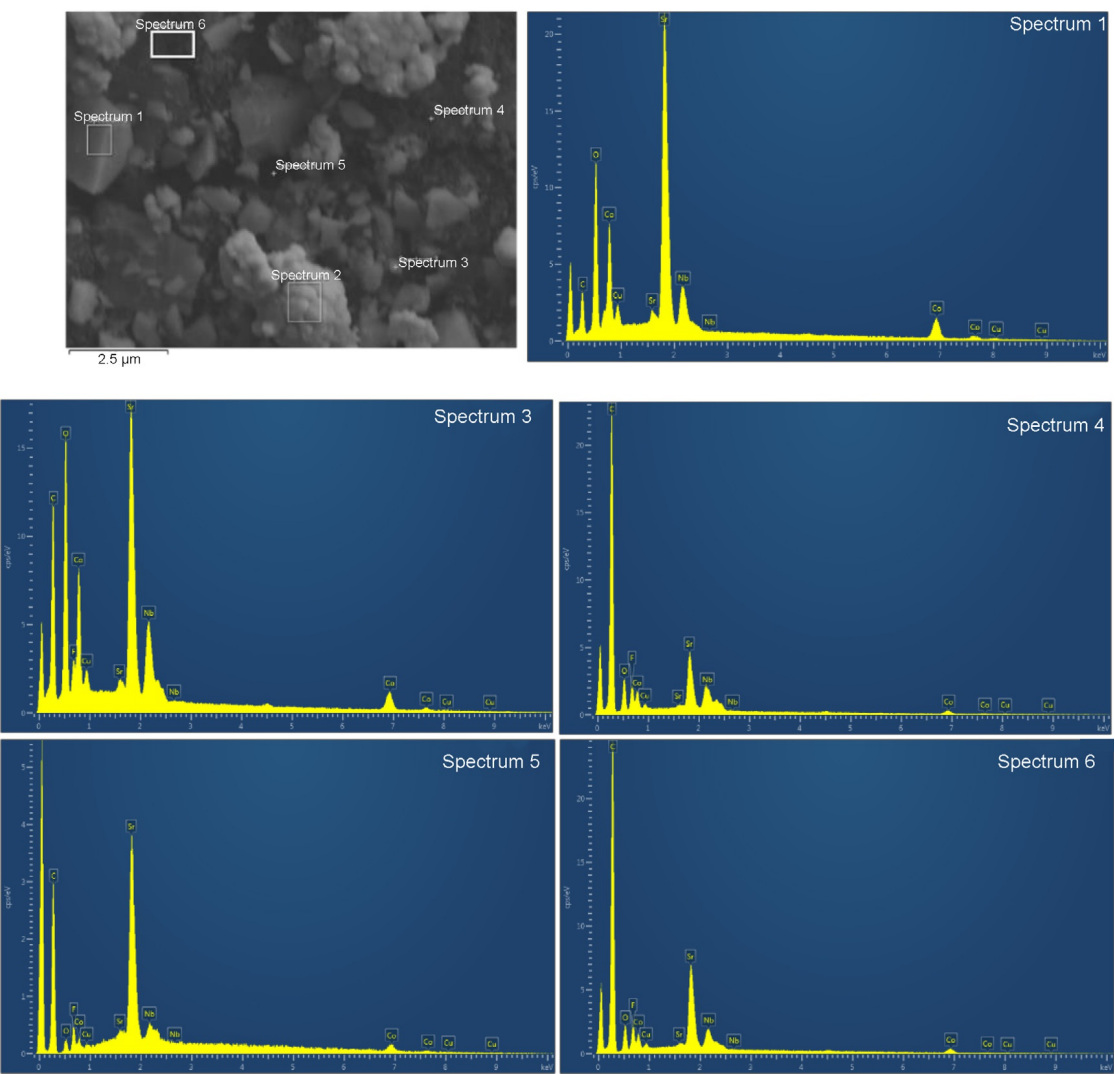
SEM observation and EDS analysis of the SrCo_0.8_Cu_0.1_Nb_0.1_O_3−*δ*_ electrode at 10 000X before the fuel cell measurements.

**Figure 7:**
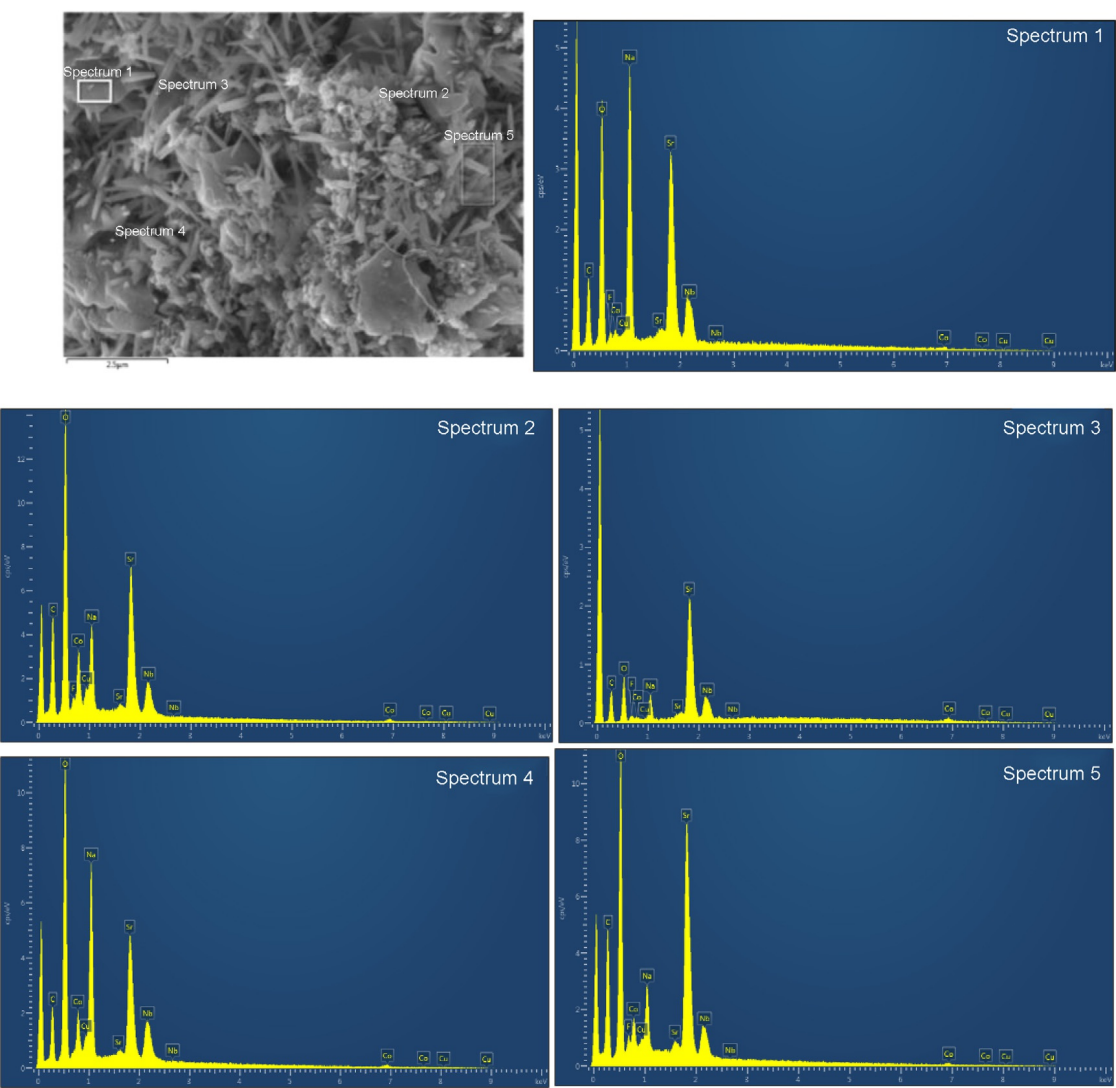
SEM observation and EDS analysis of the SrCo_0.8_Cu_0.1_Nb_0.1_O_3−*δ*_ electrode at 10 000X after fuel cell tests.

The SEM pictures of the cathode before and after the fuel cell measurements are shown in Figure 5. The SrCo_0.8_Cu_0.1_Nb_0.1_O_3−*δ*_ particles with different sizes and irregular shapes were present in the electrode before the fuel cell measurements (Figure 5 a). Further EDS analyses indicate all the particles, both large and small, are composed of SrCo_0.8_Cu_0.1_Nb_0.1_O_3−*δ*_ (Figure 6). The elements of F and C are from the PTFE binder and/or Amberlite IRA‐402(OH) resin, which are also part of the composite cathode. However, after the fuel cell test, the majority of the SrCo_0.8_Cu_0.1_Nb_0.1_O_3−*δ*_ particles were converted to shuttle‐shaped particles (Figure 5 b). EDS point analyses indicate that all these shuttle‐shaped particles are composed of Sr, Co, Cu, Nb, and O (Figure 7), which means they are still SrCo_0.8_Cu_0.1_Nb_0.1_O_3−*δ*_ combined with the XRD analysis (Figure 4 b). At 10 000 times magnification, it can be seen that the particle size of SrCo_0.8_Cu_0.1_Nb_0.1_O_3−*δ*_ was also reduced from 1 to 0.1 μm after the fuel cell measurements (Figure 6 and 7). The reason for this change in shape needs further investigation.

Figure 6 shows the EDS analysis of the electrode before fuel cell testing. According to the energy spectrum, all of the chemical elements of SrCo_0.8_Cu_0.1_Nb_0.1_O_3−*δ*_ can be observed clearly. In addition, elements of C, F, and O are visible, which come from chemicals in the ink, such as PTFE.

Figure 7 shows the EDS analysis of the cathode after fuel cell test. The EDS results have proved that the nanoscale crystal contains all of the chemical elements of SrCo_0.8_Cu_0.1_Nb_0.1_O_3–*δ*_. In addition, Na was very clearly observed as the tested fuels consist of NaOH, which may indicate fuel crossover from the anode to the cathode. Fuel cross‐over is very common in fuel cells based on polymeric electrolytes, particularly for liquid fuels. The fuel cross‐over can reduce the potential between anode and cathode, leading to decreased performance of fuel cell. This can be alleviated by adding additives to the electrolyte membrane to improve fuel cell performance.

## Conclusions

The perovskite oxide SrCo_0.8_Cu_0.1_Nb_0.1_O_3−*δ*_ was successfully synthesized using a Pechini method. X‐ray diffraction (XRD) analysis indicated that it exhibits a cubic structure with *a*=3.8806(7) Å. This study indicates that SrCo_0.8_Cu_0.1_Nb_0.1_O_3−*δ*_ is a good oxygen reduction reaction (ORR) catalyst that can be used as the cathode for a room temperature ammonia fuel cell. Reasonable open circuit voltage (OCV) and power density were observed for a direct ammonia fuel cell using a SrCo_0.8_Cu_0.1_Nb_0.1_O_3−*δ*_/C composite cathode and NiCu/C anode when ammonia solutions without the addition of NaOH were used as the fuel. An OCV of 0.19 V was observed when the ammonia concentration is as low as 0.02 m (340 ppm). The power density can be improved 7 times when adding 1 m NaOH to the fuel, and the OCV can be improved through the addition of NaOH when the ammonia concentration is lower than 1 m. Therefore, to obtain good OCV for fuel cells with low concentration ammonia in wastewater, adding NaOH is necessary, which can be achieved through the addition of alkaline waste, such as coal fly ash. The ammonia fuel cell also functions without the addition of NaOH, but will certainly take longer time to remove ammonia owing to the lower power density. Interestingly, it was found that the SrCo_0.8_Cu_0.1_Nb_0.1_O_3−*δ*_ in the cathode is converted from irregular shape into shuttle‐shape during the fuel cell measurements, which needs further investigation. As the key catalysts in this fuel cell are all non‐precious, after further development, this could be a promising technology for removal of ammonia from wastewater.

## Conflict of interest

*The authors declare no conflict of interest*.
